# Colistin Dependency among Colistin-Heteroresistant *Acinetobacter baumannii* Isolates

**DOI:** 10.3390/microorganisms10010058

**Published:** 2021-12-28

**Authors:** Hadas Kon, Amichay Hameir, Elizabeth Temkin, Alona Keren-Paz, David Schwartz, Vered Schechner, Yehuda Carmeli

**Affiliations:** 1 National Institute for Antibiotic Resistance and Infection Control, Ministry of Health, Tel Aviv 6423906, Israel; hadaskon@tlvmc.gov.il (H.K.); amichayh@tlvmc.gov.il (A.H.); lizt@tlvmc.gov.il (E.T.); alonakp@tlvmc.gov.il (A.K.-P.); davidsc@tlvmc.gov.il (D.S.); vereds@tlvmc.gov.il (V.S.); 2 Sackler Faculty of Medicine, Tel Aviv University, Tel Aviv 6997801, Israel

**Keywords:** colistin dependency, carbapenem-resistant *Acinetobacter baumannii*, colistin heteroresistance, blood culture

## Abstract

Colistin dependent (CD) isolates are dependent on colistin for optimal growth. Here we aimed to systematically determine the emergence of CD among colistin-heteroresistant carbapenem-resistant *Acinetobacter baumannii* (CRAB) isolates. We also examined the phenotypic characteristics of CD and the evolution of CD strains to overt resistance. Additionally, we examined whether detection of growth in blood cultures was impaired by CD. Heteroresistant isolates, as determined by population analysis profiling, were exposed to colistin; when the colony count with colistin was significantly higher than without, isolates were suspected to be CD. CD was confirmed by Etest and growth curves. CD strains with colistin minimum inhibitory concentrations > 2 mg/L after growth in colistin-free media were considered colistin-resistant. Of the 65 heteroresistant strains tested, eight became CD after colistin exposure. These strains attained higher colony counts and growth rates with colistin vs. without, and grew adjacent to the colistin Etest strip. CD strains exhibited increased susceptibilities to multiple antibiotics compared to their parent heteroresistant strains. All CD strains tested became colistin-resistant following growth without colistin. CD strains were detected in blood culture bottles, but time to detection was significantly prolonged compared with parent strains, suggesting that CD may lead to delay in detection of CRAB bacteremia.

## 1. Introduction

Antibiotic dependency is a phenomenon in which a bacterial strain is exposed to a specific antibiotic agent and becomes reliant on that antibiotic to reach optimal growth. The first report of antibiotic dependency was the characterization of vancomycin-dependent *Enterococcus faecalis* in 1994 [1]. A less recognized antibiotic dependency is colistin dependency (CD) in *Acinetobacter baumannii*, first reported in 2007 [2]. Colistin is a last-resort agent for treating infections c×aused by carbapenem-resistant Gram-negative bacteria including *A. baumannii* (CRAB), an extensively drug-resistant pathogen ranked in the highest category in the World Health Organization’s priority pathogens list [3].

CD is proposed to be caused by a defective outer membrane that limits bacterial growth. In the presence of colistin, the defective membrane stabilizes, and the bacteria regains the ability to grow efficiently. One described mechanism for CD is IS*Aba1* insertions in the *lpxC* gene, which encodes a lipid A biosynthetic enzyme [4]. Those mutations may result in the loss of or structural defects in lipid A that lead to a defective cell membrane and osmotic trauma. The defective cells show impaired growth, which reverses in the presence of colistin [2,4]. CD strains have high colistin minimum inhibitory concentrations (MICs) but increased susceptibility to other antibiotics, attributed to the strains’ unstable and permeable outer membrane that allows penetration of antimicrobials into the bacterial cell [4,5].

CD strains causing infection have been implicated in treatment failure and persistent bacteremia [5]. Additionally, after exposure to colistin and serial passages without colistin, CD strains may evolve and become overtly colistin-resistant [4], thus increasing the reservoir of colistin resistance.

The effect of CD on diagnosis of bacteremia is unknown. Because CD strains exhibit optimal growth in the presence of colistin, detection may be impaired: bacteremia caused by CD strains may go undetected or time to detection (TTD) may be prolonged due to the absence of colistin in the blood culture bottles.

In this study, we aimed to test for the emergence of CD among colistin-heteroresistant (HR) CRAB isolates following colistin exposure. We aimed to describe the phenotypic characteristics of CD, to examine the evolution of CD strains into overtly colistin-resistant strains, and to determine whether detection in blood cultures is impaired by CD.

## 2. Materials and Methods

### 2.1. Description of the Isolate

The sample consisted of 65 unique patient CRAB isolates (meropenem MIC > 8 mg/L) collected in 2013–2017. The isolate source was blood (*n =* 18), tracheal aspirate (*n =* 18), sputum (*n =* 14), bronchoalveolar lavage (*n =* 8), urine (*n =* 2) and unknown (*n =* 5). The isolates were classified as colistin-susceptible (colistin MIC of 0.5–2 mg/L, see methods below), and found to be HR on further testing (described below). All isolates were identified to the species level by VITEK^®^ MS (bioMérieux, Marcy l’Etoile, France). Confirmation of the species was performed by molecular genotyping, as described previously [6]. OXA typing was performed by sequencing as previously described [7]. All isolates were stored at −80 °C and sub-cultured twice on blood agar (TSA + 5% blood sheep agar, HyLabs, Rehovot, Israel) at 35 ± 2 °C before further testing. For the strains found to be CD and their parent strains Etest, growth curve, disk diffusion and blood culture assays were conducted.

### 2.2. Determination of Colistin HR

Population analysis profiling (PAP) was performed as described previously [8]. Briefly, an isolated colony was inoculated into 2 mL sterile Mueller-Hinton (MH) broth (BD, Sparks, MD, USA) and incubated overnight (18 ± 2 h) at 34–37 °C with shaking (200 rpm) to reach a stationary growth phase. The diluted bacterial culture was plated (100 μL) onto four plates containing colistin (at colistin concentrations of 1×, 2×, 4×, and 8×MIC) and on an MH colistin-free plate, all in duplicate. Colonies were counted following overnight incubation. Frequency of resistant cells was calculated as the number of colonies grown on 8×MIC plates divided by the number of colonies grown on colistin-free plates. Strains were defined as HR if colony growth was observed on plates containing colistin concentration of 8×MIC at frequencies of >10^−7^. A colistin-resistant strain (MIC 32 mg/L) and a colistin-susceptible non-HR strain (MIC 0.5 mg/L) were used as controls.

### 2.3. Broth Microdilution (BMD)

Colistin and meropenem susceptibility testing was performed by BMD. All BMD assays were performed according to ISO 20776-1 guidelines and the recommendations of the CLSI-EUCAST Polymyxin Breakpoints Working Group [9,10]. Results were read using the EUCAST BMD reading guide [11] and susceptibilities were interpreted using EUCAST 2021 breakpoints [12]. Meropenem susceptibility was tested using a commercial BMD method (customized Sensititre™ plates, ThermoFisher Scientific, Oakwood Village, OH, USA) and confirmed by a homemade BMD assay at meropenem concentrations of 0.5–64 mg/L. Quality control strains were tested as required [12,13]. Colistin susceptibility was determined using a commercial BMD method (MICRONAUT MIC-Strip colistin, MERLIN Diagnostika GmbH, Bornheim, Germany) and confirmed by a homemade BMD assay at colistin concentrations of 0.5–64 mg/L. Quality control strains were tested as required. In cases of discrepancy between results of the homemade and commercial assays, the homemade BMD test was repeated.

### 2.4. Exposure to Colistin and Determination of CD

A colony from the 8×MIC plate of the PAP assay was re-suspended in 2 mL of MH broth containing 8×MIC colistin and grown overnight at 34–37 °C with shaking. Following incubation, bacterial dilutions were streaked in duplicate on MH plates containing colistin 8×MIC and on MH colistin-free plates. The plates were incubated overnight, and colonies were counted and compared between plates containing colistin concentration of 8×MIC and colistin-free plates. Strains were suspected to be CD if Student’s *t*-test for mean CFU/mL was significantly higher in the presence of colistin compared to the absence of colistin (*p*-value of <0.05).

### 2.5. Withdrawal of Colistin and Emergence of Overt Resistance

For each CD strain, a colony from the 8×MIC plate (described above) was transferred and grown overnight in 2 mL MH broth in the absence of colistin. The bacterial culture was incubated at 34–37 °C with shaking and diluted 1:1000 with fresh MH broth every day for two additional days. The bacterial dilutions were then streaked on colistin-free MH plates and incubated overnight. The MIC of these strains was determined using BMD. If the MIC exceeded 2 mg/L (the colistin susceptibly breakpoint), the strain was considered colistin-resistant derived from colistin-dependent (CR-CD).

### 2.6. Colistin Etest

While Etest does not provide an accurate MIC for colistin, it may be helpful in visualizing CD. The test was performed according to manufacturer’s instructions. Briefly, a suspension of fresh colonies was adjusted to 0.5 McFarland, spread on an MH plate and a colistin Etest strip (bioMérieux, Marcy-l’Etoile, France) was placed on the surface. The growth pattern was observed following overnight incubation. An isolate was considered CD if heavy growth was observed proximal to the Etest strip, with scant or no growth more distally. Isolates with growth close to the strip but no growth further from the strip were considered complete CD, while isolates with growth close to the strip and scant growth further from the strip were considered partial CD [14]. CD and parent strains from these Etest plates were then streaked onto plates containing colistin (8×MIC) to verify dependency.

### 2.7. Growth Curves

Growth of CD strains and their respective parent strains was determined with and without colistin. Overnight cultures were adjusted to 0.5 McFarland and diluted in fresh MH broth to an optical density at 600 nm (OD600) of 0.008. The suspensions of each CD strain and its parent strain were inoculated in triplicates into a 96-well microplate (Cellstar 96-well plate, Greiner Bio-One, Kremsmünster, Austria) with colistin (8×MIC) and without. The plates were incubated at 34–37 °C with shaking in a microplate reader; OD600 was measured every 30 min for 24 h using Synergy HT (BioTek Instruments, Winooski, VT, USA). Differences in growth rate, maximum growth and lag time were tested using the Wilcoxon signed-rank test and *p*-values of <0.05 were considered to be statistically significant.

### 2.8. Disk Diffusion

Susceptibility to several antibiotics was assessed by disk diffusion (Oxoid Ltd., Hants, UK) [12,15]. The antibiotics included meropenem (10 µg, MEM), imipenem (10 µg, IMP), ertapenem (10 µg, ETP), ceftazidime (30 µg, CAZ), ceftriaxone (30 µg, CRO), and sulfamethoxazole/trimethoprim (1.25 µg, SXT). Susceptibilities for MEM, IMP, CAZ, CRO, and SXT were interpreted according to CLSI M100 2020 breakpoints [13]. ETP did not have a defined breakpoint for *Acinetobacter* spp.; therefore, we used the breakpoint for Gram-negative *Haemophilus influenzae* [13].

### 2.9. FT-IR Spectroscopy

Isolates were grown at 35 ± 2 °C on blood agar plates (tryptic soy agar supplemented with 5% sheep blood; Hylabs, Rehovot, Israel) for 24 h. Samples were prepared according to the IR Biotyper manufacturer’s instructions, as previously described [16]. The specimens were analyzed in three independent experiments using the IR Biotyper (Bruker, Leipzig, Germany) with the default analysis settings. Spectra were analyzed using OPUS 7.5 software (Bruker, Leipzig, Germany). Quality control was performed according to the manufacturer’s recommendations. Hierarchical cluster analysis (HCA) was generated by the OPUS 7.5 software using the Pearson correlation coefficient option. The cutoff value defining which spectra are considered to be in the same cluster was automatically generated.

### 2.10. Blood Cultures

The blood culture bottles used in this assay were BacT/Alert FA Plus (FA Plus) (bioMérieux, Durham, NC, USA). A 0.5 McFarland suspension (~2 × 10^8^ CFU/mL) of each of the CD strains and their parent strains was prepared and serial dilutions were made to reach a final concentration of 50–200 CFU/mL per blood culture bottle. Each bacterial suspension was inoculated into three separate bottles from the same diluted stock (10 mL per bottle). Each bottle contained a different colistin concentration: 0 mg/L, 0.75 mg/L or 8×MIC. The concentration of 0.75 mg/L was selected to reflect estimated therapeutic levels and 8×MIC was selected because it was the standardized concentration for all other experiments conducted in this study. The bottles were loaded into the instrument (VIRTUO Microbial Detection System, bioMérieux, Durham, NC, USA) and TTD was recorded. Following the signal of a positive blood culture, the bottles were removed and sub-cultured onto blood agar to verify the growth of the strain.

## 3. Results

**Figure 1:**
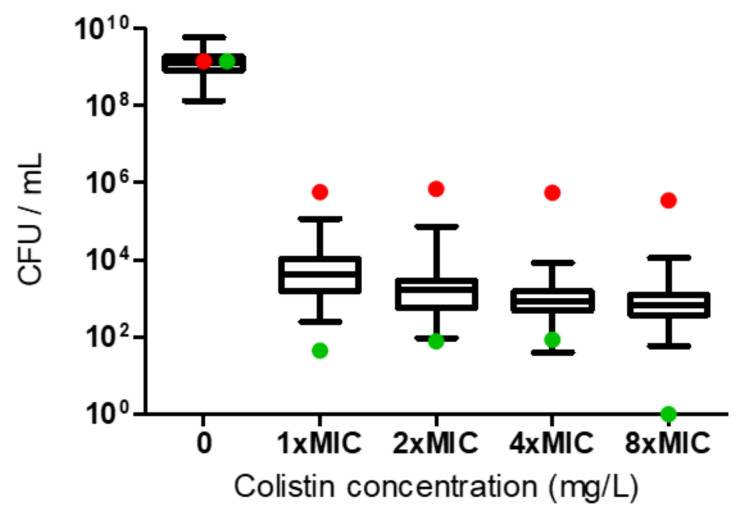
Heteroresistance (HR) determination according to population analysis profiling for the *A. baumannii* isolates. Growth of the 65 colistin-susceptible isolates at 8×MIC indicated HR. The red dots represent the colistin-resistant control, and the green dots represent the colistin-susceptible non-HR control.

The PAP assay confirmed that the 65 colistin-susceptible CRAB isolates were HR: they all grew at a colistin concentration of 8×MIC and the frequency of resistant cells ranged between 1 × 10^−7^ and 8 × 10^−6^ (Figure 1).

**Figure 2:**
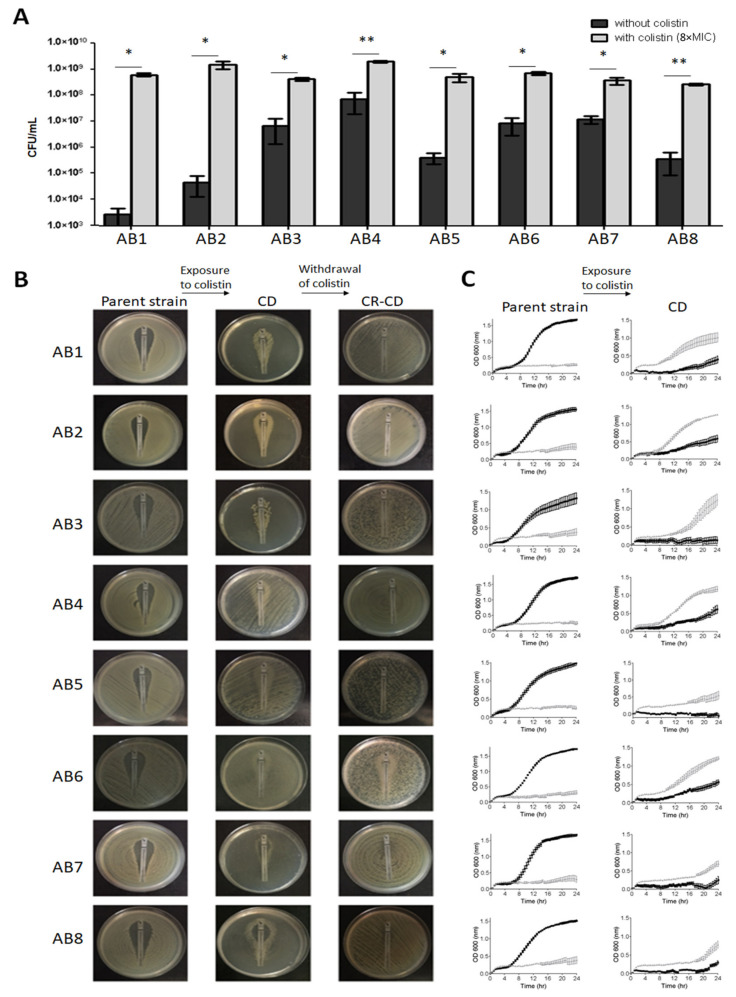
(**A**) Determination of CD. Final colony count (CFU/mL) of CD strains grown with and without colistin. Black bars represent the absence of colistin, and gray bars represent 8×MIC colistin. Results are presented as mean ± SD of two independent repeats. Mean CFU/mL was significantly higher when colistin was present than when it was absent; *p* < 0.05 for all CD strains (* *p* < 0.05, ** *p* < 0.01), as determined by Student’s *t*-test. (**B**) Colistin Etest. Etest plates of parent (left), CD (middle) and CR-CD (right) strains. (**C**) Growth curves. Growth curve of parent (**left**) and CD (**right**) strains grown with and without colistin for 24 h. Black lines represent the absence of colistin, and gray lines represent 8xMIC colistin. Results are presented as mean ± SD of three independent tests.

Following exposure to colistin, eight isolates (12.3%) had a significantly higher colony count (CFU/mL) with colistin than without colistin; these isolates were suspected to be CD and numbered AB1 through AB8 (Figure 2A). CD was confirmed using the Etest assay. Etest for the parent strains displayed inhibition zones at low MICs (Figure 2B, left panel), while CD strains exhibited growth in proximity to the Etest strip, with scant or no growth more distally (Figure 2B, middle panel). Three of the eight CD isolates presented complete CD (AB1 through AB3) and five partial CD (AB4 through AB8). Parent strains obtained from the Etest plates did not grow on colistin containing plates, whereas CD isolates did (Figure S1). After colistin withdrawal, all eight strains become overtly resistant, and had MIC values of >2 mg/L by BMD, and were thus termed CR-CD. Seven of the eight CR-CD strains grew uniformly on the entire Etest plate (Figure 2B, right panel) and one (AB6) exhibited partial CR-CD (a mixed phenotype of CD and CR-CD). None of the eight patients with CD isolates had received colistin before the sample was collected.

**Figure 3:**
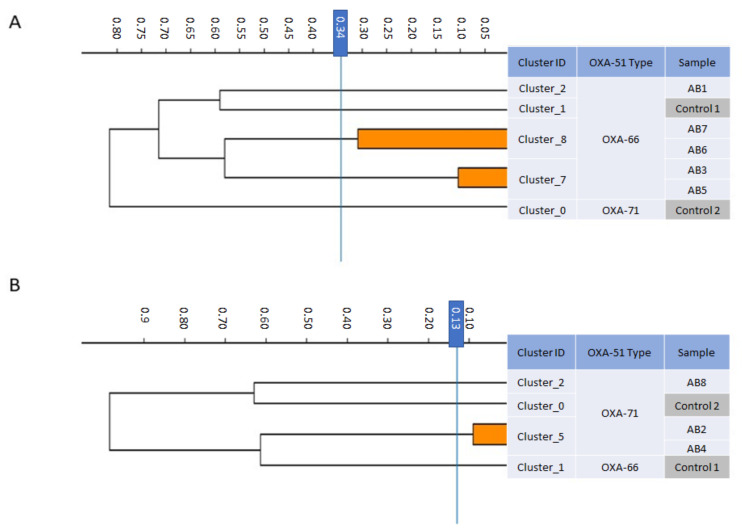
Dendrogram obtained by FT-IR biotyping of colistin-dependent *A. baumannii* isolates (*n* = 8). The blue line represents the cutoff value. Clusters composed of two of more isolates are shaded orange. (**A**) OXA-66-type strains. (**B**) OXA-71-type strains. Controls–unrelated strains from laboratory collection.

The eight CD isolates belonged to several distinct clones and clusters. Five isolates belonged to the OXA-66 clone and formed three FT-IR clusters: AB3 and AB5 formed one cluster, AB6 and AB7 formed a second cluster, and AB1 belonged to its own cluster (Figure 3A). Three isolates belonged to the OXA-71 clone and formed 2 FT-IR clusters: AB2 and AB4 formed a single cluster and AB8 belonged to its own cluster (Figure 3B). These findings suggest no recent common ancestor for the eight isolates, indicating that the CD phenotype emerged several times, independently, in unrelated strains.

Growth curves of the CD strains are shown in Figure 2C and Table 1. In the absence of colistin, CD strains exhibited slower growth rates, lower maximum growth and longer lag phases than the parent strains, while in the presence of colistin CD strains exhibited faster growth rates, higher maximum growth, and shorter lag phases compared to the parent strains. These results reflect the effect of colistin both on the CD strains and the parent strains; in the former colistin promoted growth, while in the parent strains it inhibited growth, leading to statistically significant differences in growth rates, maximum growth and lag.

**Figure 4:**
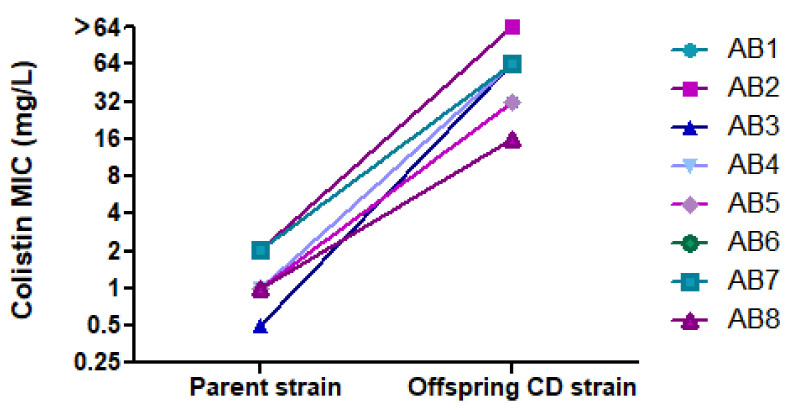
Change in colistin MIC between parent strains and their offspring CD strains.

By BMD, all parent strains were susceptible to colistin (MICs of 0.5 mg/L (*n =* 1), 1 mg/L (*n =* 4) and 2 mg/L (*n =* 3)), whereas CD offspring strains were colistin-resistant, with MICs of 16 (*n =* 1), 32 mg/L (*n =* 2), 64 mg/L (*n =* 4), and >64 mg/L (*n =* 1). Figure 4 shows the change in MIC between the parent and its offspring CD strain. When parent strains transformed into CD, they became susceptible to multiple other antibiotics to which they were previously resistant. Table 2 summarizes changes in susceptibility to 11 antibiotics.

**Figure 5:**
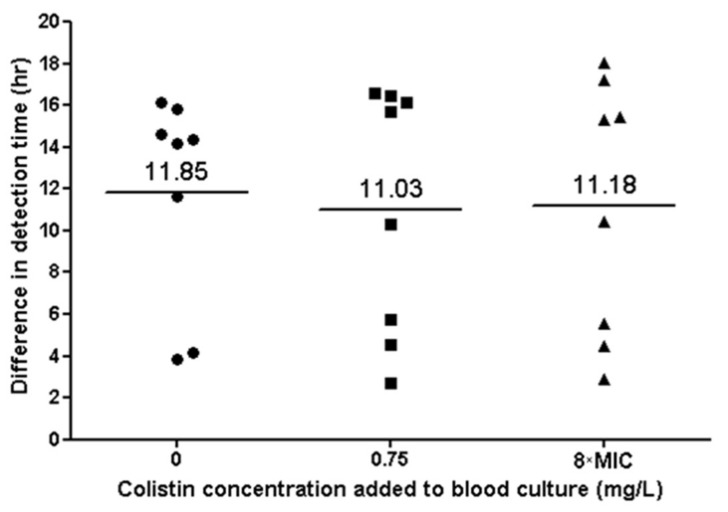
Bacterial growth in blood culture of eight colistin-dependent isolates. Each black dot represents the difference in TTD between the CD strain and its parent strain. The lines represent the average difference at each colistin concentration.

All parent and CD isolates grew in blood culture bottles. TTD was significantly shorter for the parent strains, compared to the CD strains (*p* < 0.001), with a mean difference of 11.3 h. There was no significant difference in TTD between blood culture bottles containing different colistin concentrations (0 mg/L, 0.75 mg/L and 8×MIC) (Figure 5). All tested strains grew after sub-culturing of the blood culture media on blood agar.

## 4. Discussion

Previous studies have shown that subpopulations of *A. baumannii* surviving at high colistin concentrations may exhibit the CD phenomenon [2,4,5,17]. In our study, we found eight of 65 (12.3%) colistin-HR clinical isolates to be CD. A previous study of colistin-HR *A. baumannii* or *A. calcoaceticus* complex isolates reported CD in 1/19 (5.3%) [2]. The CD isolates belonged to various clones, suggesting that the CD phenomenon is not clone specific. The multiplicity of *A. baumannii* clones in our sample is not surprising, as polyclonality has been described often [18,19,20,21].

We confirmed that CD isolates reached higher colony counts and growth rates in the presence of colistin compared to growth without colistin. These strains exhibited growth adjacent to the colistin Etest strip. We found that our CD isolates exhibited increased susceptibilities compared to their parent strains for the 11 different antibiotics tested. The clinical importance of this observation needs further study; it is possible that the permeable outer membrane of CD isolates, which allows antibiotics to penetrate the cell, may translate into new treatment strategies for CRAB infections for which treatment options are limited or not available. If confirmed, screening for CD in clinical microbiological laboratories may be of high clinical relevance.

The transformation from HR to overt resistance is assumed to be by selection of the resistant minority subpopulation in the presence of antibiotic pressure. We found that upon withdrawal of colistin pressure, all CD isolates evolved into an overtly colistin resistant phenotype. This finding may suggest that CD is an intermediate step between colistin HR and colistin resistance, at least in some strains.

An important finding of our study is that CD isolates could be detected in blood culture bottles even in the absence of colistin. However, TTD for CD strains was 11 h longer than for parent strains, suggesting that the detection of bacteremia caused by CD strains may be delayed in clinical settings. In contrast to our findings regarding growth curves, we did not observe differences in TTD for the CD strains when various colistin concentrations were added to the blood culture bottles. We hypothesize that this is due to the presence of adsorbent polymeric beads (resin) in the FA Plus bottles used, which neutralize antibiotic agents [22,23].

In conclusion, CD is found among colistin-HR CRAB isolates. It may represent an intermediate step towards an overtly resistant phenotype. CD strains are susceptible to various antibiotic agents to which their parent strains are resistant. CD may lead to delayed diagnosis of bacteremia due to impaired growth in blood culture bottles. Further studies are required to understand the link between CD and overt resistance, and to search for ways in which antibiotic susceptibilities in CD strains can be exploited to improve treatment effectiveness.

## Figures and Tables

**Table 1:** Growth parameters of parent and CD strains with and without colistin. Presented are the average difference of three independent experiments.

	CD vs. Parent Strain		With Colistin vs. Without	
	Growth in Presence of Colistin	Growth in Absence of Colistin	Growth of Parent Strain	Growth of CD Strain
Growth Rate (hr^−1^)	0.06 *	−0.11 *	−0.13 **	0.04 *
Maximal Growth (lnOD)	0.67 *	−1.24 *	−1.25 **	0.65 *
Lag time (hr)	−13.41 *	11.05 *	16.58 **	−7.89 *

* *p* < 0.05, ** *p* < 0.01.

**Table 2:** Antibiotic resistance profiles. Inhibition zones (mm) by disk diffusion of parent (P), colistin-dependent (CD), and colistin resistant-colistin dependent (CR-CD) strains.

	Meropenem (MEM)			Imipenem (IMP)			Ertapenem (ETP)			Ceftazidime (CAZ)			Ceftriaxone (CRO)			Sulfamethoxazole Trimethoprim (SXT)		
	P	CD	CR-CD	P	CD	CR-CD	P	CD	CR-CD	P	CD	CR-CD	P	CD	CR-CD	P	CD	CR-CD
AB1	0	39	35	0	35	33	0	31	33	0	34	24	0	35	21	18	36	31
AB2	0	41	41	10	36	39	0	37	34	0	27	26	0	28	22	14	30	32
AB3	9	37	38	10	35	36	0	33	34	0	40	30	0	35	34	0	0	0
AB4	0	33	37	0	31	23	0	31	26	0	29	23	0	31	20	0	34	22
AB5	8	36	34	9	35	33	0	33	30	0	30	30	0	31	30	0	0	0
AB6	0	30	37	0	37	36	0	27	35	0	40	39	0	45	31	19	45	38
AB7	0	40	11	0	38	11	0	40	0	0	36	0	0	36	0	16	34	0
AB8	11	45	13	0	45	11	0	45	0	11	>45	0	0	45	0	0	0	0

Yellow indicates susceptible, orange intermediate, and red resistant.

## Data Availability

The data presented in this study are available on request from the corresponding author.

## Supplementary Materials

The following are available online at https://www.mdpi.com/article/10.3390/microorganisms10010058/s1, Figure S1: Verification of colistin dependency by Etest.
