# Supplementary material for: Colistin Dependency among Colistin-Heteroresistant Acinetobacter baumannii Isolates
Source: Microorganisms. 2021 Dec 28;10(1):58. doi: 10.3390/microorganisms10010058 (PMC8780235; doi:10.3390/microorganisms10010058)
Supplement: Supplementary file 1 [file microorganisms-10-00058-s001.zip › microorganisms-1487642-supplementary.pdf]

Supplementary Materials

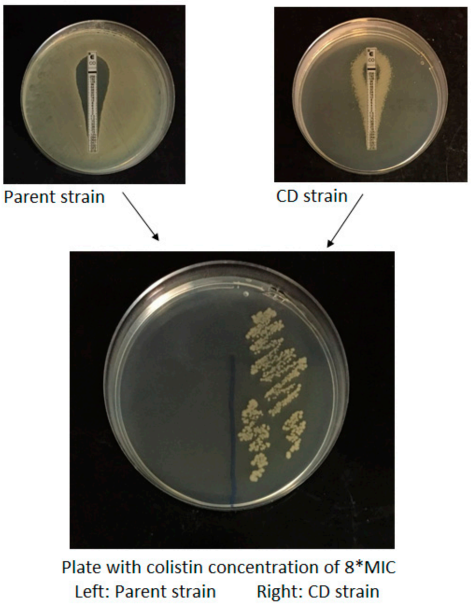

Figure S1. Verification of colistin dependency by Etest. Parent strain (left) and CD strain (right) from Etest plates were streaked onto a colistin-containing plate (on left and right sides of plate, respectively). Displayed is a representative strain (AB2); similar results were obtained for all CD strains.
